# Is it the Time to Move Towards Coronary Computed Tomography Angiography-Derived Fractional Flow Reserve Guided Percutaneous Coronary Intervention? The Pros and Cons

**DOI:** 10.2174/1573403X19666230119115228

**Published:** 2023-07-05

**Authors:** Mohammadbagher Sharifkazemi, Zahra Hooshanginezhad, Arezou Zoroufian, Kamran Shamsa

**Affiliations:** 1 Division of Cardiology, Nemazee Hospital, Shiraz University of Medical Sciences, Shiraz, Iran;; 2 Division of Cardiology, Tehran Heart Center, Tehran University of Medical Sciences, Tehran, Iran;; 3 Division of Cardiology, David Geffen School of Medicine, University of California, Los Angeles, CA, 90095, USA

**Keywords:** Invasive coronary angiography, fractional flow reserve, myocardial, coronary computed tomography angiography, treatment decision-making, coronary artery disease

## Abstract

Coronary artery disease is the leading cause of mortality worldwide. Diagnosis is conventionally performed by direct visualization of the arteries by invasive coronary angiography (ICA), which has inherent limitations and risks. Measurement of fractional flow reserve (FFR) has been suggested for a more accurate assessment of ischemia in the coronary artery with high accuracy for determining the severity and decision on the necessity of intervention. Nevertheless, invasive coronary angiography-derived fractional flow reserve (ICA-FFR) is currently used in less than one-third of clinical practices because of the invasive nature of ICA and the need for additional equipment and experience, as well as the cost and extra time needed for the procedure. Recent technical advances have moved towards non-invasive high-quality imaging modalities, such as magnetic resonance, single-photon emission computed tomography, and coronary computed tomography (CT) scan; however, none had a definitive modality to confirm hemodynamically significant coronary artery stenosis. Coronary computed tomography angiography (CCTA) can provide accurate anatomic and hemodynamic data about the coronary lesion, especially calculating fractional flow reserve derived from CCTA (CCTA-FFR). Although growing evidence has been published regarding CCTA-FFR results being comparable to ICA-FFR, CCTA-FFR has not yet replaced the invasive conventional angiography, pending additional studies to validate the advantages and disadvantages of each diagnostic method. Furthermore, it has to be identified whether revascularization of a stenotic lesion is plausible based on CCTA-FFR and if the therapeutic plan can be determined safely and accurately without confirmation from invasive methods. Therefore, in the present review, we will outline the pros and cons of using CCTA-FFR *vs*. ICA-FFR regarding diagnostic accuracy and treatment decision-making.

## 1 INTRODUCTION

The prevalence of Cardiovascular disease (CVD), including coronary artery disease (CAD), heart failure (HF), stroke, and hypertension, has been reported at 49.2% in adults over 20 years of age by the American Heart Association (AHA) in March 2021 annual statistical update report. This report was based on 2015-2018 data collected by the National Health and Nutrition Examination Survey (NHANES). The prevalence of CVD, excluding hypertension, has been reported at 9.3% [1]. CVD is among the top causes of death worldwide, responsible for at least one-third of the all-cause mortality rate, mainly attributed to ischemic heart disease and stroke [2]. The age-standardized death rate of CVD varies among nations depending on the overall incidence of CVD in that region, determined by the risk factors in that population, such as obesity, physical inactivity, hyperlipidemia, hypertension, diabetes mellitus, chronic kidney disease, and environmental risks such as air pollution, which vary prominently among different regions [3]. Another critical determinant of CVD-related mortality rate is attributed to the diagnostic and therapeutic strategies used to manage coronary artery disease (CAD), investment in which has resulted in a decreasing trend in CVD-related mortality during the past 25 years in countries with a high sociodemographic index, despite the plateau or gradual decline in CVD-related mortality rate in most regions of the world [4, 5]. The implication of underlying CVD has also been magnified over the past two years during the Coronavirus disease 2019 (COVID-19) pandemic, which has resulted in over 6 million deaths (as of April 2022), as the presence of CVD increases the vulnerability to severe outcome from COVID-19 infection, including the risk of hospitalization and death [6, 7].

The conventional therapeutic approaches for CVD include medical therapies (such as statins, beta-blockers, nitrates, and antiplatelets), rarely lytic therapy in the setting of an acute event, and most commonly, invasive evaluation for determination of the severity of CAD and potential options for revascularization. Accurate diagnosis is the primary step for appropriate therapeutic choice [8].

Parameters used for diagnosis of CAD are categorized into suggestive and definite diagnostic tools; the former includes primary clinical diagnostic measures, such as clinical symptoms and signs, new changes on electrocardiogram (ECG), and serum biomarkers, by which acute myocardial infarction (AMI) can be promptly suspected. Several tree models have been suggested to increase a better estimation of these parameters [9, 10]. More importantly, definite measures to determine the luminal proportion of stenosis are of great importance, according to which the therapeutic approach can be chosen [11]. There are different diagnostic modalities to estimate the degree of stenosis, including invasive coronary angiography (ICA) as well as noninvasive imaging techniques such as cardiac computed tomography (CT), positron emission tomography (PET), hybrid PET/CT, and conventional single-photon emission CT (SPECT) [12]. The fractional flow reserve (FFR) is defined as the discrepancy of the coronary artery pressure between the proximal and distal sites to a significant stenotic lesion in the presence of pharmacologic vasodilatation [13]. FFR is the most accurate diagnostic measure to determine the need for revascularization in hemodynamically significant coronary artery lesions. The FFR is traditionally calculated by using a pressure wire through invasive coronary angiography; however, later on, it is evaluated through invasive coronary angiography-derived fractional flow Reserve (ICA-FFR) without the need for passing the pressure wire [14]. Considering the invasive nature of ICA-FFR new methods for evaluating the discrepancy between anatomical and functional behavior of significant coronary lesions were established, such as the noninvasive CT-derived fractional flow reserve (CT-FFR) [15]. As the accuracy of diagnostic tools is changing each day with the advance in technology, it is required to update the literature in this regard; therefore, in the present review, we aimed to identify the risks and benefits of the conventional invasive coronary angiography-derived fractional flow Reserve(ICA-FFR) *versus* coronary computed tomography angiography (CCTA) derived fractional flow reserve (FFR) (CCTA-FFR) measurement, to determine if a change in clinical practice is warranted.

## 2 CONVENTIONAL INVASIVE CORONARY ANGIOGRAPHY (ICA)/ PERCUTANEOUS CORONARY INTERVENTION

ICA, introduced in 1958, was one of the main accomplishments in cardiology. It has been used as the standard and conventional diagnostic method of CAD ever since because of the reproducible visualization of CAD and it’s severity on angiographic images obtained during direct injection of contrast agent into the coronary arteries [16]. To the growing evidence from the physicians’ observations, several changes have been made to the initial ICA, including obtaining digital images, reducing the contrast volume and the number of wires used, and developing quantitative measurements, which have resulted in more accurate images, fewer complications, higher success rates, and reduced procedural time [16, 17].

Another important advantage of ICA is the capability of simultaneous percutaneous coronary intervention (PCI), which has shown high success rates (above 95%) [18]. PCI has also shown promising results in patients with chronic total occlusion compared to medical therapy alone [19-21]. Early ICA has also reduced all-cause and cardiac mortality in patients with acute coronary events [22]. Comparing the results of PCI with coronary artery bypass grafting (CABG) has shown PCI (using drug-eluting stents) as safe and effective as CABG in patients with left main coronary artery disease at low surgical risk with lower rates of repeated revascularization required after CABG [23, 24]; however, CABG is considered the treatment of choice in patients with multivessel coronary artery disease with higher survival, lower major cardiovascular events (MACE), and repeated revascularization rate [25, 26]. Moreover, the success rate of patients’ revascularization using PCI depends on several factors, such as the CAD’s type and severity, as well as the type of technique, instrument, and drugs used during and after the PCI [27].

On the one hand, numerous benefits have been proposed for ICA and PCI, making them the gold standard for diagnosis and method of choice in the management of most patients with CAD; but after half a century after being introduced, several consequential limitations have been noted [28], such as the fact that ICA is a laminography technique and might miss tubular lesions [29]. Another critical limitation of ICA includes the inter-and intra-observer bias because of different interpretations of coronary angiograms that cannot be altered because of the nature of this method [30]. A further limitation of ICA is related to the remarkable influence of factors affecting the flow dynamics of a vessel, other than its point narrowing, as well as the fact that the decision of revascularization should not only be based on the angiographic severity but also the hemodynamic significance of the lesion [31]. For this reason, FFR measurement has been suggested as a functional measurement of stenosis severity, which subverts the limitation of ICA in the pure anatomic assessment of stenosis severity [32].

## 3 INVASIVE CORONARY ANGIOGRAPHY DERIVED FRACTIONAL FLOW RESERVE (ICA-FFR)

Measurement of ICA-FFR was introduced in 1993 by Pijles and colleagues, based on the concept of coronary flow reserve about two decades earlier, as the ratio of distal intracoronary pressure to aortic pressure during maximum hyperemia and minimum microvascular resistance induced by dilator drugs [33]. Later, introducing a floppy-tipped guidewire with a pressure sensor added resistance to trans-stenotic flow and facilitated this measurement [34]. The pressure wire–derived FFR became widely used after that, owing to its independence from the baseline flow and relative simplicity and cost-effectiveness for treatment decision-making compared with ICA [35-37]. However, some have suggested the higher [38] or comparable [39] diagnostic accuracy of ICA-FFR, compared with wire-based, which unnecessarily imposes the patients to the wire pressure. In this method, coronary stenosis is measured by the reduction of perfusion pressure induced by the viscous and expansion losses of the narrowed vessel, calculated by the Poiseuille’s law and Bernoulli’s equation, and reported by the fraction from the normal value of 1.0 in every patient and every vessel [40]. Poiseuille’s law shows that the coronary flow equals the difference between baseline and maximum perfusion pressure divided by the capillary hemodynamic resistance: (P2−P1)π × r(radius)^4^/8 × η(viscosity) × l(length) [41]. Bernoulli’s equation also calculates the pressure gradient (Δ*p*) by the sum of Δ*P*_convective_, Δ*P*_constriction_, Δ*P*_diffusive_, and Δ*P*_expansion_ [42].

Several benefits have been considered for FFR. First, it can discriminate between patients with flow-limiting CAD, requiring coronary revascularization, and patients without, who benefit most from medical therapy [37, 43]. A large-scale study showed that the use of FFR could change the management of 43% of patients compared with pure ICA assessment [44]. Furthermore, the measurement of FFR during maximum hyperemia is unaffected by the patient’s hemodynamics, including heart rate and blood pressure, making ICA-FFR a highly reproducible assessment method [45]. Also, as both antegrade and collateral flow are considered in the distal pressure, the collateral blood supply is taken into account during FFR measurement [46]. The initial study on FFR suggested values <0.75 for the diagnosis of inducible ischemia (with a specificity of 100%) and values above 0.80 for its ruling out (with a sensitivity of 90%) (33), while values ≤0.80 are currently considered significant obstruction requiring revascularization [47]. Therefore, the lesion’s severity significantly affects the diagnostic accuracy of FFR, as clinical justification is difficult and controversial within the “grey zone” of FFR, between 0.75 and 0.85, in cases with intermediate lesions [48]. Therefore, it is suggested to reconsider the FFR cut-off used for the treatment decision based on the complexity of the coronary lesion [49].

Although several trials, including FAME, DEFER, and PRIME trials, have reported favorable results considering the clinical outcome for the decision of revascularization based on FFR [50], this measurement has several limitations [51, 52]. An important limitation of this measurement, besides the points mentioned earlier, such as the operator-dependent variability of its diagnostic accuracy [53], is related to the factors that influence FFR, such as hyperemic microvascular resistance, the size of perfusion territory, minimal lumen diameter, and lesion length, which can all influence FFR [51, 52]. Low perfusion pressures and hemodynamic factors, like tachycardia, can affect microvascular resistance, decreasing diastolic duration. Because of the reduced arteriolar caliber induced by reduced distending pressure, there is a non-linear relationship between coronary pressure and flow that violates the main assumption of the FFR test. To mitigate this limitation, it has been suggested to consider coronary flow reserve (CFR) and the index of microvascular resistance during this procedure, as well [54, 55]. Researchers also tried to develop other software and computational models to estimate the fluid dynamics, such as reduced-order and steady-state models, which reduced the consumed time. Still, these models showed limited diagnostic values [56, 57]. In addition to the limitations, ICA-FFR has several risks due to the invasive nature of the procedure, including local vascular injury, AMI, cholesterol emboli, conduction disturbances, cerebrovascular complications, nephropathy, dissection/perforation of great vessels and coronary artery, and death [28].

Considering the limitations, risks, and complications of ICA-FFR, several imaging techniques have been suggested for non-invasive assessment of CAD, including coronary ultrasound, nuclear myocardial perfusion imaging using single-photon emission computed tomography, contrast- and stress-induced magnetic resonance imaging (MRI); however, each of the proposed methods has its limitations and disadvantages, considered beneficiary only in a specific subgroup of patients, and not comparable to ICA-FFR [58, 59]. Nevertheless, recent evidence has suggested CCTA-FFR as an appropriate non-invasive tool for quantified assessment of coronary stenosis with higher diagnostic accuracy for vessel-specific ischemia, compared with other non-invasive methods [60], with the capability to provide vessel-specific anatomic information, despite other non-invasive methods [61]. However, it has not yet replaced ICA in the clinical setting.

## 4 CORONARY COMPUTED TOMOGRAPHY ANGIOGRAPHY (CCTA)

Since the introduction of the 16-detector row CCTA in 2005, a growing literature has been dedicated to the diagnostic accuracy of this test. Early trials suggested a high diagnostic accuracy for detection of ≥70% stenosis by CCTA in low-risk patients (without known CAD) [62-64], as well as a high negative predictive value (NPV) for CCTA (95% in general; 93% per-lesion NPV and 97% per-patient NPV) that suggest CCTA as an appropriate alternative to ICA [65, 66]. Furthermore, CCTA has the advantage of plaque identification over ICA, although lower specificity rates have been reported for myocardial ischemia [67]. One of the differences among the studies is attributed to the alterations in techniques and radiation doses used for CCTA over the years. Although CCTA has shown better diagnostic accuracy and prognostic outcomes *vs*. other non-invasive imaging modalities [59], it has the limitation of not considering the hemodynamic significance of the lesion, just like ICA, as far as the anatomical indices are only measured during CCTA for estimating CAD severity, including the luminal diameter stenosis, area stenosis, minimum lumen diameter, and minimum lumen area [31]. Accordingly, several methods have been suggested to overcome these limitations, such as dual-layer spectral detectors [68] and static and dynamic CT myocardial perfusion imaging techniques, which evaluate myocardial contrast material uptake. Still, they have been restricted to the studied centers because of the high radiation dose and performance difficulty [69]. Additional analyses, such as transluminal attenuation gradient and corrected models of coronary opacification, could also not overcome this limitation of CCTA, as well, because of the several factors that influence image quality and the time-consuming nature of these methods [70]. The fractional myocardial mass has been used only in the preliminary study and has not been validated yet [71]. A recent study in the United Kingdom also shows that performing CCTA in patients with recent-onset chest pain could omit the need for further investigation in most patients. The ICA is still used in almost half of such cases currently as part of management, with no resulting revascularization [72], which suggests moving from ICA to CCTA as a safer and more viable alternative. CCTA-FFR is the novel method introduced for the anatomic-functional mismatched measurements of CCTA without additional imaging or medications required, which has several advantages over ICA and, thus the potential of replacing ICA in the future.

## 5 CORONARY COMPUTERIZED TOMOGRAPHY ANGIOGRAPHY-DERIVED FFR (CCTA-FFR)

The preliminary studies on CCTA-FFR confirmed the high diagnostic accuracy of this measurement, although they were performed with insufficient image qualities [73, 74]. In 2013, Taylor and colleagues developed CCTA-FFR by three-dimensional modeling using semi-automatic coronary segmentation algorithms by a single low-dose scan [75]. For this purpose, the anatomic model of the patient’s aortic and epicardial coronary arteries is created, and maximal hyperemia is simulated using the complex Navier-Stokes equations to calculate FFR, considering blood as an incompressible Newtonian fluid with a constant viscosity within the coronary arteries [75]. Based on the review of studies, the most common formulation is:


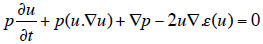


where “u” is calculated based on the rigid domain (Ω1) × velocity, “p” by Ω1 × the pressure, and “ε (u)” by the strain rate tensor [76].

The only US Food and Drug Administration-approved process to utilize this technology is Heart Flow (Redwood City, CA, USA), which takes data received from CCTA and utilizes its propriety algorithm to provide complete CCTA-FFR. Recently, machine learning approaches have been suggested that use a multilayer neural network architecture from an extensive database of coronary anatomies and hemodynamic conditions, which showed a sensitivity of 81%, a specificity of 84%, and an accuracy of 83% in the early study with a significant decrease in the computation time [77].

## 6 DIAGNOSTIC ACCURACY OF CCTA-FFR *VS*. CCTA AND ICA-FFR

Although the ICA-FFR is the gold standard for the selection of patients for revascularization in cases of discrepancy between anatomical findings and patients’ symptoms, recent studies revealed a high accuracy of CCTA-FFR in this condition [78-80]. Several clinical trials and meta-analyses of studies have estimated the diagnostic accuracy of CCTA-FFR *vs*. CCTA and ICA-FFR. The first version of CCTA-FFR was performed by Koo and colleagues in 2011, the DISCOVER-FLOW study (Diagnosis of Ischemia-Causing Stenoses Obtained *via* Non-invasive Fractional Flow Reserve), on 159 vessels in 103 patients with known or suspected CAD that showed a sensitivity of 91.4% and specificity of 39.6% with a diagnostic accuracy of 58.5% for CCTA-FFR and confirmed the improved specificity and diagnostic accuracy of CCTA-FFR over CCTA alone [73]. The second trial, DeFACTO (Determination of Fractional Flow Reserve by Anatomic Computed Tomographic Angiography), was a multicentral study on 407 vessels in 252 patients with known or suspected CAD that used the second-generation CCTA-FFR algorithm (version 1.2) and confirmed the higher diagnostic accuracy, sensitivity and NPV of CCTA-FFR in the diagnosis of ischemia in lesions with intermediate stenosis severity, compared with CCTA (74). Min and co-workers also reported that although the per-patient diagnostic accuracy of CCTA-FFR did not reach the prespecified goal, it improved the diagnosis of hemodynamically significant obstructive CAD compared with CCTA alone [81]. The low image quality and other technical/methodological shortcomings were other concerns in the two initial trials. Therefore, further studies focused on automated image processing methods to better identify boundaries and use physiological models for microvasculature resistance to achieve more accurate results. The following version mandated nitroglycerine and β-blockers and minimized image noise [31]. The subsequent trial, the NXT trial (Analysis of Coronary Blood Flow Using CT Angiography: Next Steps), a multicentral study on 484 vessels in 254 patients, showed higher per-patient and per-vessel specificity and positive predictive value (PPV) compared with ICA with lower sensitivity rates [82]. These three clinical trials used 64-sectioned CT. Other single-center studies, which used either 64- or 128-sectioned CT on a sample size <100 patients, also confirmed the higher specificity and PPV of CCTA-FFR than ICA [66, 83].

Meta-analysis studies have also evaluated the diagnostic accuracy of CCTA-FFR by pooled analysis. In 2015, Gonzales and colleagues reported per-patient specificity of 72% and PPV of 70% for CCTA-FFR, which was higher than that of CCTA (43% and 56%, respectively) without difference in sensitivity rates (94% *vs*. 92%, respectively). There were also no differences in sensitivity and specificity between CCTA and CCTA-FFR in the per-vessel study [84]. Although per-vessel diagnostic accuracy rates are a bit lower than per-patient rates, the superiority of CCTA-FFR *vs*. CCTA is maintained [84].

In another meta-analysis of five studies in 2015 on 706 patients and 1165 vessels, per-patient sensitivity and specificity of CCTA-FFR were reported at 90% and 72%, and per-lesion rates at 83% and 78%, respectively [85]. In 2018, Agashti and others performed a meta-analysis of 17 studies (1294 patients and 2194 vessels). They reported per-patient sensitivity and specificity of CCTA-FFR at 83% and 72%, respectively, and per-vessel rates at 85% and 76%, respectively [86]. The difference in diagnostic accuracy rates of the studies can be related to the different inclusion criteria for selecting studies, as single-center studies are predisposed to selection bias. The pooled analysis (of five studies, 908 vessels, 536 patients) by Cook and colleagues showed that the cut-off considered for CCTA-FFR also affects the reported diagnostic accuracy; as reported, the overall diagnostic accuracy of 82% was obtained by CCTA-FFR values between 0.63 and 0.83, while values <0.53 showed a diagnostic accuracy of 95% and values >0.93 a diagnostic accuracy of 98% [87]. Diagnostic accuracy of 100% was reported by other researchers for CCTA-FFR values <0.7, while it decreased to 73% in values >0.8 [88]. Liu *et al*. used a machine learning algorithm for CCTA-FFR as an alternative to diagnostic ICA for selecting the proper candidates for revascularization. CCTA-FFR learning algorithm Using the cut-off value of 0.80 resulted in 72% prevention of unnecessary ICA performed within two years in patients with >50% stenosis [89].

There are also some limitations for CCTA-FFR, including the lower diagnostic accuracy of this tool in intermediate lesions [90, 91] and a high rate of false-positive in terminal vessels [92]. Still, the superiority of CCTA-FFR *vs*. CCTA alone has also been confirmed in intermediate lesions [93] and distal-to-the-lesion sites [92]. The impact of calcification has also been considered in the subgroup analysis of the NXT trial, indicating lower diagnostic accuracy rates in patients with calcified lesions (Agatston scores of 416–3599) [94]. Advanced technology, such as dual-energy CT and calcium removal by material decomposition imaging, has been suggested for increased diagnostic accuracy of calcified arteries [95]. Therefore, the effect of other complex lesions on the diagnostic accuracy of CCTA-FFR requires more studies. Some have also suggested further computational methods, such as AccuFFR-CCTA, to increase the diagnostic accuracy of this test and omit the risk of invasive tests [96]. However, this method can also not eliminate the limitations mentioned above.

## 7 CLINICAL IMPLICATION OF CCTA-FFR *VS*. ICA-FFR

The decision of treatment (revascularization or medical therapy) is the imaging method's main goal. Therefore, besides the diagnostic accuracy, it must be determined whether CCTA-FFR is an appropriate tool for this decision. Based on the results of the PROMISE (PROspective Multicenter Imaging Study for Evaluation of Chest Pain) trial on patients who were referred with stable chest pain within 90 days after CCTA, diagnosis of severe stenosis and ischemia by FFR values ≤0.8 was reported as a better predictor of revascularization and MACE, compared with ICA and superior to CTA alone, and resulted in 44% decrease in ICA, showing no obstructive CAD (less than 50% stenosis), which suggests CCTA-FFR as a gatekeeper for an efficient referral to ICA [97]. Also, in the PLATFORM trial (Prospective Longitudinal Trial of FFRCT: Outcome and Resource Impacts), observation of obstructive CAD on ICA within 90 days after CCTA showed no difference in MACE and CCTA-FFR resulted in the cancellation of ICA in 61% of patients [98]. Others have also confirmed that utilization of CCTA-FFR reduces the need for ICA and, thus, its complications [99]. The study by Curzen and colleagues showed a change in the treatment strategy of 36% of patients by CCTA-FFR, 18% decreased vessel revascularization and 5% PCI, and 23% change in the optimal medical therapy; the main factor for this change was the differences between the severity of the lesion assessed by CCTA and CCTA-FFR [100]. Kim and colleagues used a virtual coronary stenting computational model in 44 patients (48 lesions). The lesions were measured before and after the intervention using CCTA-FFR; the results showed minor differences in FFR derived from ICA and CCTA before and after the intervention, with the excellent agreement [101]. Furthermore, in patients with stable angina, CCTA-FFR reduced the use of ICA, although it did not improve the clinical outcomes compared with standard clinical care [102]. Also, in candidates for transthoracic aortic valve replacement, CCTA-FFR could predict MACE [103]. Therefore, CCTA-FFR has been considered an appropriate tool for identifying the correct target lesion and optimal stent size. However, further investigations are required to determine the safety and feasibility of applying non-invasive methods solely in real-world clinical practice.

In a pilot study, five cardiac surgeons were randomized to decide the treatment plan of 20 patients based on either CCTA or ICA, considering FFR used for calculation of SYNTAX score (by subtracting non-flow limiting stenoses (CCTA-FFR >0.80) from the CCTA-derived anatomical SYNTAX score). The results showed excellent agreement on the number of bypass grafts required (correlation coefficient of 0.77) [104]. Further, the randomization of heart teams, including a cardiac surgeon and radiologist, to decide the treatment of patients with *de novo* three-vessel or left main coronary stenosis for PCI or CABG based on either CCTA-FFR or ICA confirmed a high agreement between the two methods (Cohen’s kappa 0.82), 80% on revascularization strategy, while the use of this novel tool could change the treatment plan in 7% of patients with the multi-vessel disease (from 92.2% to 78.8%) [105]. In another study, Andreini and co-workers evaluated the effect of CCTA-FFR on the treatment decision of patients (either PCI or CABG), decided by two heart teams based on CCTA or ICA-FFR (randomly assigned). The results showed changed treatment by CCTA-FFR in 7% of patients, vessel selection for revascularization in 12%, and reclassification from intermediate and high to low SYNTAX score tertile in 14% of patients [106].

The first study that addressed the safety and feasibility of using CCTA-FFR alone for treatment decision-making was the FASTTRACK CABG trial (2020), which evaluated the use of CCTA-FFR for planning surgical revascularization in patients with complex coronary artery disease; the surgeons reported that this modality was safely applicable for management of patients without ICA in 84% of cases [107]. In addition to the confirmed usefulness of CCTA-FFR in diagnosing and managing CAD, compared with ICA-FFR [108], other advantages have also been elaborated for this novel method, including reduced cost and improved patients’ quality of life [109, 110]. Nevertheless, this method is also not exempt from limitations, elaborated further below.

## 8 LIMITATIONS OF CCTA-FFR

Several limitations have been pointed out for CCTA-FFR, which result from the post-processing calculation of the score, as well as lack of global access to its calculation. In addition, CCTA-FFR is also affected by the limitations of CT imaging, such as image quality, artifacts, misalignment, motion artifact, beam hardening, and image noise, which have been minimized by the recent suggestion of administrating medications during the procedure to reduce heart rate and heart rate variability (β-blockers) and dilate the coronary arteries (sublingual nitroglycerin), as well as the suggestion of more advanced scanners [95, 111]. Furthermore, there are inherent limitations related to the FFR calculation, such as cases with intermediate lesions, calcification, non-ischemia-related lesions, and other complex coronary lesions. Other limitations include the different responses of microcirculation to vasodilators and physiological conditions affecting fluid density and viscosity among patients, compromising the accuracy of the results of CCTA-FFR. There are also several conditions, the effects of which have not been identified on FFR, such as severe anemia and reduced blood viscosity; hence further studies are required under these conditions.

## CONCLUSION

Although some of the limitations of ICA have been mitigated by ICA-FFR measurement, this gold standard diagnostic test for CAD still has several drawbacks. CCTA-FFR is a novel approach to the assessment of CAD lesions. The previous studies have provided positive results for its high diagnostic accuracy, including higher specificity and PPV, which suggests CCTA-FFR may be a superior diagnostic modality to ICA-FFR (with similar sensitivity rates). Clinical outcomes using this innovative tool could result in more appropriate treatment decisions and decreased need for ICA-FFR, thus reducing the risks associated with the invasive procedure. Based on the present review, we suggest CCTA-FFR as an effective and accurate diagnostic tool; however, it has its limitations, as outlined. Despite promising data that has been provided in this review, there remains insufficient evidence, due to the lack of large randomized clinical trials, to conclude whether this novel non-invasive method, CCTA-FFR, can replace the ICA-FFR in the management of patients with suspected CAD. Further studies are warranted to assess the safety and feasibility of planning coronary artery revascularization solely based on CCTA-FFR.

## LIST OF ABBREVIATIONS

AMI: Acute Myocardial Infarction
CABG: Coronary Artery Bypass Grafting
CAD: Coronary Artery Disease
CT: Computed Tomography
CVD: Cardiovascular Disease
HF: Heart Failure
AHA: American Heart Association
NHANES: National Health and Nutrition Examination Survey
DeFACTO: Determination of Fractional Flow Reserve by Anatomic Computed Tomographic Angiography
DISCOVER-FLOW: Diagnosis of Ischemia-Causing Stenoses Obtained *via* Non-Invasive Fractional Flow Reserve
ECG: Electrocardiogram
FFR: Fractional Flow Reserve
ICA: Invasive Coronary Angiography
ICA-FFR: Invasive Coronary Angiography-Fractional Flow Reserve
MACE: Major Cardiovascular Events
MRI: Magnetic Resonance Imaging
PCI: Percutaneous Coronary Intervention
PLATFORM: Prospective Longitudinal Trial of FFRCT: Outcome and Resource Impacts

## References

[1] Dornquast C., Kroll L.E., Neuhauser H.K., Willich S.N., Reinhold T., Busch M.A. (2016). Regional differences in the prevalence of cardiovascular disease.. Dtsch. Arztebl. Int..

[2] Bhatnagar P., Wickramasinghe K., Williams J., Rayner M., Townsend N. (2015). The epidemiology of cardiovascular disease in the UK 2014.. Heart.

[3] Mensah G.A., Roth G.A., Fuster V. (2019). The global burden of cardiovascular diseases and risk factors: 2020 and beyond.. J. Am. College Cardiol..

[4] Roth G.A., Johnson C., Abajobir A. (2017). Global, regional, and national burden of cardiovascular diseases for 10 causes, 1990 to 2015.. J. Am. Coll. Cardiol..

[5] Townsend N., Wilson L., Bhatnagar P., Wickramasinghe K., Rayner M., Nichols M. (2016). Cardiovascular disease in Europe: Epidemiological update 2016.. Eur. Heart J..

[6] Bansal M. (2020). Cardiovascular disease and COVID-19.. Diabetes Metab. Syndr..

[7] Nishiga M., Wang D.W., Han Y., Lewis D.B., Wu J.C. (2020). COVID-19 and cardiovascular disease: From basic mechanisms to clinical perspectives.. Nat. Rev. Cardiol..

[8] Joseph P., Leong D., McKee M. (2017). Reducing the global burden of cardiovascular disease, part 1: The epidemiology and risk factors.. Circ. Res..

[9] Ghiasi M.M., Zendehboudi S., Mohsenipour A.A. (2020). Decision tree-based diagnosis of coronary artery disease: CART model.. Comput. Methods Programs Biomed..

[10] Joloudari J.H., Hassannataj Joloudari E., Saadatfar H. (2020). Coronary artery disease diagnosis; ranking the significant features using a random trees model.. Int. J. Environ. Res. Public Health.

[11] Bonaca M.P., Wiviott S.D., Braunwald E. (2012). American college of cardiology/American heart association/European society of cardiology/world heart federation universal definition of myocardial infarction classification system and the risk of cardiovascular death: Observations from the triton-timi 38 trial (trial to assess improvement in therapeutic outcomes by optimizing platelet inhibition with prasugrel-thrombolysis in myocardial infarction 38).. Circulation.

[12] Di Carli M.F., Hachamovitch R. (2007). New technology for noninvasive evaluation of coronary artery disease.. Circulation.

[13] Gao Z., Wang X., Sun S. (2020). Learning physical properties in complex visual scenes: An intelligent machine for perceiving blood flow dynamics from static CT angiography imaging.. Neural Netw..

[14] Collet C., Onuma Y., Sonck J. (2018). Diagnostic performance of angiography-derived fractional flow reserve: A systematic review and Bayesian meta-analysis.. Eur. Heart J..

[15] Liu X., Wang Y., Zhang H. (2019). Evaluation of fractional flow reserve in patients with stable angina: Can CT compete with angiography?. Eur. Radiol..

[16] Ryan T.J. (2002). The coronary angiogram and its seminal contributions to cardiovascular medicine over five decades.. Circulation.

[17] Wang K.T., Chen C.Y., Chen Y.T. (2014). Improving success rates of percutaneous coronary intervention for chronic total occlusion at a rural Hospital in East Taiwan.. Int. J. Gerontol..

[18] Sondagur A.R., Wang H., Cao Y., Lin S., Li X. (2014). Success rate and safety of coronary angiography and angioplasty via radial artery approach among a Chinese population.. J. Invasive Cardiol..

[19] Nikolakopoulos I., Vemmou E., Karacsonyi J. (2020). Latest developments in chronic total occlusion percutaneous coronary intervention.. Expert Rev. Cardiovasc. Ther..

[20] Khanra D., Mishra V., Jain B. (2020). Percutaneous coronary intervention provided better long term results than optimal medical therapy alone in patients with chronic total occlusion: A meta-analysis.. Indian Heart J..

[21] Lee S.H., Cho J.Y., Kim J.S. (2020). A comparison of procedural success rate and long-term clinical outcomes between in-stent restenosis chronic total occlusion and de novo chronic total occlusion using multicenter registry data.. Clin. Res. Cardiol..

[22] Kosyakovsky L.B., Austin P.C., Ross H.J. (2021). Early invasive coronary angiography and acute ischaemic heart failure outcomes.. Eur. Heart J..

[23] Nerlekar N., Ha F.J., Verma K.P. (2016). Percutaneous coronary intervention using drug-eluting stents versus coronary artery bypass grafting for unprotected left main coronary artery stenosis: A meta-analysis of randomized trials.. Circ. Cardiovasc. Interv..

[24] Gao L., Liu Y., Sun Z., Wang Y., Cao F., Chen Y. (2017). Percutaneous coronary intervention using drug-eluting stents versus coronary artery bypass graft surgery in left main coronary artery disease an updated meta-analysis of randomized clinical trials.. Oncotarget.

[25] Thuijs D.J.F.M., Kappetein A.P., Serruys P.W. (2019). Percutaneous coronary intervention versus coronary artery bypass grafting in patients with three-vessel or left main coronary artery disease: 10-year follow-up of the multicentre randomised controlled SYNTAX trial.. Lancet.

[26] Spadaccio C., Benedetto U. (2018). Coronary Artery Bypass Grafting (CABG) vs. Percutaneous Coronary Intervention (PCI) in the treatment of multivessel coronary disease: quo vadis—a review of the evidences on coronary artery disease.. Ann. Cardiothorac. Surg..

[27] Baykan A.O., Gür M., Acele A. (2016). Predictors of successful percutaneous coronary intervention in chronic total coronary occlusions.. Postepy Kardiol. Interwencyjnej.

[28] Tavakol M., Ashraf S., Brener S.J. (2012). Risks and complications of coronary angiography: A comprehensive review.. Glob. J. Health Sci..

[29] (2015). Kočka V. The coronary angiography - An old-timer in great shape.. Cor Vasa.

[30] Garg S., Girasis C., Sarno G. (2010). The SYNTAX score revisited: A reassessment of the SYNTAX score reproducibility.. Catheter. Cardiovasc. Interv..

[31] Tesche C., De Cecco C.N., Albrecht M.H. (2017). Coronary CT angiography–derived fractional flow reserve.. Radiology.

[32] Elgendy I.Y., Conti C.R., Bavry A.A. (2014). Fractional flow reserve: An updated review.. Clin. Cardiol..

[33] Pijls N.H., van Son J.A., Kirkeeide R.L., De Bruyne B., Gould K.L. (1993). Experimental basis of determining maximum coronary, myocardial, and collateral blood flow by pressure measurements for assessing functional stenosis severity before and after percutaneous transluminal coronary angioplasty.. Circulation.

[34] De Bruyne B., Paulus W.J., Vantrimpont P.J., Sys S.U., Heyndrickx G.R., Pijls N.H.J. (1993). Transstenotic coronary pressure gradient measurement in humans: In vitro and in vivo evaluation of a new pressure monitoring angioplasty guide wire.. J. Am. Coll. Cardiol..

[35] Nam J., Briggs A., Layland J. (2015). Fractional Flow Reserve (FFR) versus angiography in guiding management to optimise outcomes in non-ST segment elevation myocardial infarction (FAMOUS-NSTEMI) developmental trial: cost-effectiveness using a mixed trial- and model-based methods.. Cost Eff. Resour. Alloc..

[36] van Nunen L.X., Zimmermann F.M., Tonino P.A.L. (2015). Fractional flow reserve versus angiography for guidance of PCI in patients with multivessel coronary artery disease (FAME): 5-year follow-up of a randomised controlled trial.. Lancet.

[37] Zhang D., Lv S., Song X. (2015). Fractional flow reserve versus angiography for guiding percutaneous coronary intervention: A meta-analysis.. Heart.

[38] Gong Y., Zheng B., Yi T. (2021). Coronary angiography‐derived contrast fractional flow reserve.. Catheter. Cardiovasc. Interv..

[39] Chang C.C., Lee Y.H., Chuang M.J. (2021). Agreement between invasive wire-based and angiography-based vessel fractional flow reserve assessment on intermediate coronary stenoses.. Front. Cardiovasc. Med..

[40] Achenbach S., Rudolph T., Rieber J. (2017). Performing and interpreting fractional flow reserve measurements in clinical practice: An expert consensus document.. Interv. Cardiol..

[41] Faes T.J.C., Meer R., Heyndrickx G.R., Kerkhof P.L.M. (2020). Fractional flow reserve evaluated as metric of coronary stenosis - a mathematical model study.. Front. Cardiovasc. Med..

[42] Huo Y., Svendsen M., Choy J.S., Zhang Z.D., Kassab G.S. (2012). A validated predictive model of coronary fractional flow reserve.. J. R. Soc. Interface.

[43] Tonino P.A.L., De Bruyne B., Pijls N.H.J. (2009). Fractional flow reserve versus angiography for guiding percutaneous coronary intervention.. N. Engl. J. Med..

[44] Van Belle E., Rioufol G., Pouillot C. (2014). Outcome impact of coronary revascularization strategy reclassification with fractional flow reserve at time of diagnostic angiography: Insights from a large French multicenter fractional flow reserve registry.. Circulation.

[45] Lim W.H., Koo B.K., Nam C.W. (2015). Variability of fractional flow reserve according to the methods of hyperemia induction.. Catheter. Cardiovasc. Interv..

[46] Adiputra Y., Chen S.L. (2015). Clinical relevance of coronary fractional flow reserve: art-of-state.. Chin. Med. J..

[47] Melikian N., De Bondt P., Tonino P. (2010). Fractional flow reserve and myocardial perfusion imaging in patients with angiographic multivessel coronary artery disease.. JACC Cardiovasc. Interv..

[48] Petraco R., Sen S., Nijjer S. (2013). Fractional flow reserve-guided revascularization: Practical implications of a diagnostic gray zone and measurement variability on clinical decisions.. JACC Cardiovasc. Interv..

[49] Mohdnazri S.R., Keeble T.R., Sharp A.S.P. (2016). Fractional flow reserve: Does a cut-off value add value?. Interv. Cardiol..

[50] Weerts J., Pustjens T., Amin E. (2021). Long‐term outcome after deferred revascularization due to negative fractional flow reserve in intermediate coronary lesions.. Catheter. Cardiovasc. Interv..

[51] Jeremias A., Kirtane A.J., Stone G.W. (2017). A test in context: fractional flow reserve: Accuracy, prognostic implications, and limitations.. J. Am. Coll. Cardiol..

[52] Crystal G., Klein L. (2015). Fractional flow reserve: Physiological basis, advantages and limitations, and potential gender differences.. Curr. Cardiol. Rev..

[53] Lal K., Gosling R., Ghobrial M. (2021). Operator-dependent variability of angiography-derived fractional flow reserve and the implications for treatment.. Eur Heart J Digit Health.

[54] Garcia D., Harbaoui B., van de Hoef T.P. (2019). Relationship between FFR, CFR and coronary microvascular resistance – Practical implications for FFR-guided percutaneous coronary intervention.. PLoS One.

[55] Berry C. (2014). Fractional flow reserve, coronary flow reserve and the index of microvascular resistance in clinical practice.. EuroIntervention.

[56] Morris P.D., Ryan D., Morton A.C. (2013). Virtual fractional flow reserve from coronary angiography: modeling the significance of coronary lesions: results from the VIRTU-1 (VIRTUal Fractional Flow Reserve From Coronary Angiography) study.. JACC Cardiovasc. Interv..

[57] Morris P.D., Silva Soto D.A., Feher J.F.A. (2017). Fast virtual fractional flow reserve based upon steady-state computational fluid dynamics analysis: Results from the VIRTU-Fast study.. JACC Basic Transl. Sci..

[58] Heo R., Nakazato R., Kalra D., Min J.K. (2014). Noninvasive imaging in coronary artery disease.. Semin. Nucl. Med..

[59] Hecht H.S., Narula J., Fearon W.F. (2016). Fractional flow reserve and coronary computed tomographic angiography.. Circ. Res..

[60] Driessen R.S., Danad I., Stuijfzand W.J. (2019). Comparison of coronary computed tomography angiography, fractional flow reserve, and perfusion imaging for ischemia diagnosis.. J. Am. Coll. Cardiol..

[61] Dewey M., Siebes M., Kachelrieß M. (2020). Clinical quantitative cardiac imaging for the assessment of myocardial ischaemia.. Nat. Rev. Cardiol..

[62] Budoff M.J., Dowe D., Jollis J.G. (2008). Diagnostic performance of 64-multidetector row coronary computed tomographic angiography for evaluation of coronary artery stenosis in individuals without known coronary artery disease: results from the prospective multicenter ACCURACY (Assessment by Coronary Computed Tomographic Angiography of Individuals Undergoing Invasive Coronary Angiography) trial.. J. Am. Coll. Cardiol..

[63] Miller J.M., Rochitte C.E., Dewey M. (2008). Diagnostic performance of coronary angiography by 64-row CT.. N. Engl. J. Med..

[64] Meijboom W.B., Meijs M.F.L., Schuijf J.D. (2008). Diagnostic accuracy of 64-slice computed tomography coronary angiography: A prospective, multicenter, multivendor study.. J. Am. Coll. Cardiol..

[65] Chow B.J.W., Abraham A., Wells G.A. (2009). Diagnostic accuracy and impact of computed tomographic coronary angiography on utilization of invasive coronary angiography.. Circ. Cardiovasc. Imaging.

[66] Renker M., Schoepf U.J., Wang R. (2014). Comparison of diagnostic value of a novel noninvasive coronary computed tomography angiography method versus standard coronary angiography for assessing fractional flow reserve.. Am. J. Cardiol..

[67] Min J.K., Shaw L.J., Berman D.S. (2010). The present state of coronary computed tomography angiography a process in evolution.. J. Am. Coll. Cardiol..

[68] Xu C., Yi Y., Han Y. (2021). Incremental improvement of diagnostic performance of coronary CT angiography for the assessment of coronary stenosis in the presence of calcium using a dual-layer spectral detector CT: Validation by invasive coronary angiography.. Int. J. Cardiovasc. Imaging.

[69] Varga-Szemes A., Meinel F.G., De Cecco C.N., Fuller S.R., Bayer R.R., Schoepf U.J. (2015). CT myocardial perfusion imaging.. AJR Am. J. Roentgenol..

[70] Henriksson L., Woisetschläger M., Alfredsson J. (2020). The transluminal attenuation gradient does not add diagnostic accuracy to coronary computed tomography.. Acta Radiol..

[71] Kim H.Y., Lim H.S., Doh J.H. (2016). Physiological severity of coronary artery stenosis depends on the amount of myocardial mass subtended by the coronary artery.. JACC Cardiovasc. Interv..

[72] Morgan-Hughes G., Williams M.C., Loudon M. (2021). Downstream testing after CT coronary angiography: time for a rethink?. Open Heart.

[73] Koo B.K., Erglis A., Doh J.H. (2011). Diagnosis of ischemia-causing coronary stenoses by noninvasive fractional flow reserve computed from coronary computed tomographic angiograms. Results from the prospective multicenter DISCOVER-FLOW (Diagnosis of Ischemia-Causing Stenoses Obtained via Noninvasive Fractional Flow Reserve) study.. J. Am. Coll. Cardiol..

[74] Nakazato R., Park H.B., Berman D.S. (2013). Noninvasive fractional flow reserve derived from computed tomography angiography for coronary lesions of intermediate stenosis severity: Results from the DeFACTO study.. Circ. Cardiovasc. Imaging.

[75] Taylor C.A., Fonte T.A., Min J.K. (2013). Computational fluid dynamics applied to cardiac computed tomography for noninvasive quantification of fractional flow reserve: scientific basis.. J. Am. Coll. Cardiol..

[76] Arbia G., Vignon-Clementel I.E., Hsia T.Y., Gerbeau J.F. (2016). Modified Navier-Stokes equations for the outflow boundary conditions in hemodynamics.. Eur. J. Mech. BFluids.

[77] Itu L, Rapaka S, Passerini T (2016). A machine-learning approach for computation of fractional flow reserve from coronary computed tomography.. J Appl Physiol (1985).

[78] Westra J., Andersen B.K., Campo G. (2018). Diagnostic performance of in‐procedure angiography‐derived quantitative flow reserve compared to pressure‐derived fractional flow reserve: The favor ii europe‐japan study.. J. Am. Heart Assoc..

[79] Budoff M.J., Nakazato R., Mancini G.B.J. (2016). CT angiography for the prediction of hemodynamic significance in intermediate and severe lesions.. JACC Cardiovasc. Imaging.

[80] Pijls N.H.J. (2013). Fractional flow reserve to guide coronary revascularization.. Circ. J..

[81] Min J.K., Leipsic J., Pencina M.J. (2012). Diagnostic accuracy of fractional flow reserve from anatomic CT angiography.. JAMA.

[82] Nørgaard B.L., Leipsic J., Gaur S. (2014). Diagnostic performance of noninvasive fractional flow reserve derived from coronary computed tomography angiography in suspected coronary artery disease: the NXT trial (Analysis of Coronary Blood Flow Using CT Angiography: Next Steps).. J. Am. Coll. Cardiol..

[83] Kruk M, Wardziak Ł, Demkow M, *et al* (2016). Workstation-based calculation of CTA-based FFR for intermediate stenosis.. JACC Cardiovasc. Imaging.

[84] Gonzalez J.A., Lipinski M.J., Flors L., Shaw P.W., Kramer C.M., Salerno M. (2015). Meta-analysis of diagnostic performance of coronary computed tomography angiography, computed tomography perfusion, and computed tomography-fractional flow reserve in functional myocardial ischemia assessment versus invasive fractional flow reserve.. Am. J. Cardiol..

[85] Deng S.B., Jing X.D., Wang J. (2015). Diagnostic performance of noninvasive fractional flow reserve derived from coronary computed tomography angiography in coronary artery disease: A systematic review and meta-analysis.. Int. J. Cardiol..

[86] Agasthi P., Kanmanthareddy A., Khalil C. (2018). Comparison of computed tomography derived fractional flow reserve to invasive fractional flow reserve in diagnosis of functional coronary stenosis: A meta-analysis.. Sci. Rep..

[87] Cook C.M., Petraco R., Shun-Shin M.J. (2017). Diagnostic accuracy of computed tomography–derived fractional flow reserve: A systematic review.. JAMA Cardiol..

[88] Nørgaard B.L., Hjort J., Gaur S. (2017). Clinical use of coronary CTA–derived FFR for decision-making in stable CAD.. JACC Cardiovasc. Imaging.

[89] Liu X., Mo X., Zhang H., Yang G., Shi C., Hau W.K. (2021). A 2-year investigation of the impact of the computed tomography–derived fractional flow reserve calculated using a deep learning algorithm on routine decision-making for coronary artery disease management.. Eur. Radiol..

[90] Yang D.H., Kim Y.H., Roh J.H. (2017). Diagnostic performance of on-site CT-derived fractional flow reserve versus CT perfusion.. Eur. Heart J. Cardiovasc. Imaging.

[91] Coenen A., Rossi A., Lubbers M.M. (2017). Integrating CT myocardial perfusion and CT-FFR in the work-up of coronary artery disease.. JACC Cardiovasc. Imaging.

[92] Cami E., Tagami T., Raff G. (2021). Importance of measurement site on assessment of lesion-specific ischemia and diagnostic performance by coronary computed tomography angiography-derived fractional flow reserve.. J. Cardiovasc. Comput. Tomogr..

[93] Baumann S., Renker M., Hetjens S. (2016). Comparison of coronary computed tomography angiography-derived vs. invasive fractional flow reserve assessment: Meta-analysis with subgroup evaluation of intermediate stenosis.. Acad. Radiol..

[94] Nørgaard B.L., Gaur S., Leipsic J. (2015). Influence of coronary calcification on the diagnostic performance of CT angiography derived FFR in coronary artery disease: A substudy of the NXT trial.. JACC Cardiovasc. Imaging.

[95] Andreini D., Pontone G., Mushtaq S. (2015). Diagnostic accuracy of rapid kilovolt peak–switching dual-energy CT coronary angiography in patients with a high calcium score.. JACC Cardiovasc. Imaging.

[96] Jiang W., Pan Y., Hu Y. (2021). Diagnostic accuracy of coronary computed tomography angiography-derived fractional flow reserve.. Biomed. Eng. Online.

[97] Lu M.T., Ferencik M., Roberts R.S. (2017). Noninvasive FFR derived from coronary CT angiography.. JACC Cardiovasc. Imaging.

[98] Douglas P.S., Pontone G., Hlatky M.A. (2015). Clinical outcomes of fractional flow reserve by computed tomographic angiography-guided diagnostic strategies vs. usual care in patients with suspected coronary artery disease: the prospective longitudinal trial of FFR CT: outcome and resource impacts study.. Eur. Heart J..

[99] Rabbat M., Leipsic J., Bax J. (2020). Fractional flow reserve derived from coronary computed tomography angiography safely defers invasive coronary angiography in patients with stable coronary artery disease.. J. Clin. Med..

[100] Curzen N.P., Nolan J., Zaman A.G., Nørgaard B.L., Rajani R. (2016). Does the routine availability of CT–derived FFR influence management of patients with stable chest pain compared to CT angiography alone? The FFRCT RIPCORD study.. JACC Cardiovasc. Imaging.

[101] Kim K.H., Doh J.H., Koo B.K. (2014). A novel noninvasive technology for treatment planning using virtual coronary stenting and computed tomography-derived computed fractional flow reserve.. JACC Cardiovasc. Interv..

[102] Curzen N., Nicholas Z., Stuart B. (2021). Fractional flow reserve derived from computed tomography coronary angiography in the assessment and management of stable chest pain: the FORECAST randomized trial.. Eur. Heart J..

[103] Aquino G.J., Abadia A.F., Schoepf U.J. (2022). Coronary CT fractional flow reserve before transcatheter aortic valve replacement: Clinical outcomes.. Radiology.

[104] Sonck J., Miyazaki Y., Collet C. (2019). Feasibility of planning coronary artery bypass grafting based only on coronary computed tomography angiography and CT-derived fractional flow reserve: A pilot survey of the surgeons involved in the randomized SYNTAX III Revolution trial.. Interact. Cardiovasc. Thorac. Surg..

[105] Collet C., Onuma Y., Andreini D. (2018). Coronary computed tomography angiography for heart team decision-making in multivessel coronary artery disease.. Eur. Heart J..

[106] Andreini D., Modolo R., Katagiri Y. (2019). Impact of fractional flow reserve derived from coronary computed tomography angiography on heart team treatment decision-making in patients with multivessel coronary artery disease: Insights from the SYNTAX III REVOLUTION trial.. Circ. Cardiovasc. Interv..

[107] Kawashima H., Pompilio G., Andreini D. (2020). Safety and feasibility evaluation of planning and execution of surgical revascularisation solely based on coronary CTA and FFR CT in patients with complex coronary artery disease: Study protocol of the FASTTRACK CABG study.. BMJ Open.

[108] Andreini D., Mushtaq S., Conte E. (2020). The usefulness of cardiac CT integrated with FFRCT for planning myocardial revascularization in complex coronary artery disease: A lesson from SYNTAX studies.. Cardiovasc. Diagn. Ther..

[109] Douglas P.S., De Bruyne B., Pontone G. (2016). 1-year outcomes of FFRCT-guided care in patients with suspected coronary disease: The PLATFORM study.. J. Am. Coll. Cardiol..

[110] Hlatky M.A., De Bruyne B., Pontone G. (2015). Quality-of-life and economic outcomes of assessing fractional flow reserve with computed tomography angiography: PLATFORM.. J. Am. Coll. Cardiol..

[111] Cho I, Elmore K, ó Hartaigh B, *et al* (2015). Heart-rate dependent improvement in image quality and diagnostic accuracy of coronary computed tomographic angiography by novel intracycle motion correction algorithm.. Clin. Imaging.

